# Supplementary material for: Chromothripsis during telomere crisis is independent of NHEJ, and consistent with a replicative origin
Source: Genome Res. 2019 May;29(5):737–49. doi: 10.1101/gr.240705.118 (PMC6499312; doi:10.1101/gr.240705.118)
Supplement: Supplemental Material [file supp_gr.240705.118_Supplemental_file_1.zip › contigs/annotated_contigs/DB115/contig.2.DB115_length_361_mean_cov_4.57617728532.docx]

**DB115_length_361_mean_cov_4.57617728532**

```
CCAACAGGGTGAAACCCTGTCTCTACTAAAAATACAAAAAATTAGGTTGATGTGGTGGCACGCGACTGTAGTCCCAGGTACTTTATAGG
 >chr10:113427213-113427445 + E=5e-100
CTGAGGCAGGAGAATCACTTGAACCCAGGAGACAGAGGTTGTAGTGAGCCAAGATTATTCCATTGCACTCCAGCCTGGGCAACAAGAGC

AAAACTCCATCTCAAAAACAAAACAAAACAAAACAAAACAAAACAAACAAACAAA|CAAAAACT|TATATATATATATATATATATATG
                                                                >chr10:113427968-113428
AGTTTATTAAGTATTAACTCACACGATCACAAGGTCCTACAATAGGCCGTCTGCAGGCTGAGGAGCAAGGAGAGCCAGTCTGAGGTTCT
088 + E=8e-61
AAAACTG
```
